# Benefits of Environmentally Friendly Plaster on Mechanical Properties When Combined with Polyester Resin and Hardener Are Examined under Compression and Tension

**DOI:** 10.3390/polym16192847

**Published:** 2024-10-09

**Authors:** Mohammed A. Albadrani, Ahmed D. Almutairi

**Affiliations:** 1 Department of Mechanical Engineering, College of Engineering, Qassim University, Buraydah 51452, Saudi Arabia; 2 Department of Civil Engineering, College of Engineering, Qassim University, Buraydah 51452, Saudi Arabia; ah.almutairi@qu.edu.sa

**Keywords:** compression test, eco-friendly, sustainability, tensile test, mechanical properties, polyester

## Abstract

Recently, plaster has gained increasing attention as a mechanical and environmentally friendly option and is an effective alternative to traditional cement products. Additionally, polyester has an effective impact on the mechanical properties of materials, in addition to being one of the most environmentally friendly materials. However, studies are still ongoing to reach the best ratios of polyester resin, polyester hardener, and gypsum plaster that can improve mechanical properties. This research aims to investigate the impact of these components at various ratios (30%, 45%, and 60%) of gypsum plaster weight on the mechanical properties of plaster material. This study is carried out by conducting compression and tensile tests for three ratios, which are considered among the most important mechanical tests according to their applications. In addition, the environmental emissions resulting from the three different ratios of plaster are evaluated to determine their environmental impact. This study found that the largest ratio (30%) was the most effective from an economic and mechanical point of view, while achieving lower carbon emissions compared to the other ratios, which enhances the trend towards achieving the environmental goals of the Kingdom of Saudi Arabia’s Vision 2030 to reach zero emissions. This study is highly significant both in terms of scientific research and practical application across a range of industries, since it integrates the enhancement of material performance with the achievement of environmental sustainability requirements.

## 1. Introduction

### Background

The world’s natural resources are running out, which raises serious questions about how future global demand will be met. The next problem is to keep future demand and sustainable supply in balance. Researchers have focused on environmentally compatible materials because of growing ecological and socioeconomic awareness, excessive petroleum resource usage, and new environmentally friendly policies, particularly in European countries. The use of particulate fillers to improve the desired properties of polymers or to lower the cost of composites has generated significant interest in the enhanced performance of polymers and their composites in various applications, such as in industrial and structural fields [1].

Polymer concrete has been used in the construction, transport, and electrical industries. Different products are produced, for example, those prefabricated for drainage and cladding of building facades. In the electrical sector, polymer concrete is used as support in bushing pieces for electrical insulation, replacing the conventional insulators such as ceramics and glass. Applications of polymer concrete depend on the physicochemical characteristics of the aggregates and resin, where the latter depends on the storage time after its production. The authors are proposing the solution to combine plaster and polymer in order to solve the problem of storage for a long period, where initial manufacturing properties are lost over time.

Polyester resin is a thermosetting polymer with several uses that is both economical and adaptable. Plaster, on the other hand, is a gypsum-based substance that cures quickly and is very user-friendly. These substances can be mixed to create composites with better mechanical qualities. However, while choosing polyester resin for a particular project, one must take into account both its benefits—like its adaptability and chemical resistance—and drawbacks—like its brittleness and low heat resistance. In polymer concrete, polyester resin plays a key role as the binding agent in place of cement [2,3,4,5].

Polyester resins are considered “eco-friendly”, even though they are derived from crude oil in certain cases, due to the low emissions produced during use, since the polyester resin is manufactured in a way that complies with environmental sustainability standards [4,5,6,7].

Polyester-based polymer concrete provides several advantages over traditional cement-based concrete, such as faster curing, increased chemical resistance, decreased permeability, and better mechanical properties. It can be used in construction, electrical/electronics, precast parts, industrial flooring, coatings, and composites. Among the drawbacks include its price and susceptibility to temperature and environmental conditions. Because of their strength, stiffness, and minimal moisture absorption, thermosetting polymers known as unsaturated polyester resins, or UPRs, are widely used in both the military and industrial sectors [6,7,8].

Understanding these aspects of polyester resin in polymer concrete is essential for its effective use in various construction and industrial applications. Several studies are presented to investigate the effect of polyester resins and their different applications.

A recent publication has provided an outline of the latest research findings, as well as the expanding market for bio-based thermosetting polymers. Despite current research efforts, only a few commercially available bio-based thermosetting resin systems were identified in the most recent market survey. Nonetheless, they were typically suitable for a wide range of applications and processing techniques (up to 75%) [9]. When another study looked at the flexural, hardness, and tensile strength of a composite material reinforced with echinates polyester and resin fibers, it concluded that the composite material with 0 orientation and a 70% echinates fiber to 30% polyester ratio had the highest tensile strength (60.60 MPa), flexural strength (96.8 MPa), and hardness values (44.54 HRA). It was determined that 70% fiber at 0 orientation echinates-fiber-reinforced composites are good substitutes for synthetic materials [10]. They use five types of powders from commonly available fillers, including sand (S), pit sand (PS), plaster of Paris (POP), white cement (WC), and black cement (BC). The UPR/BC-10 and UPR/BC-20 samples had the highest percentages of water absorption, being 6.202 and 5.07%, respectively. The distribution, size, and combination of the filler and polymer, in addition to the filler concentration, were found to have an impact on the attributes of CPCs, according to the study’s findings [11]. One study found that Luffa Fiber (LF) inclusions affected the mechanical and thermal properties of composites made of unsaturated polyester resin [12]. It was found that, when LF inclusion increases, the mechanical characteristics gradually improve. The maximum values of hardness and compressive strength were 81.5 N/mm^2^ and 43.9 MPa, respectively, while the ideal impact resistance was 2.34 KJ/m^2^. Simultaneously, there was a noticeable decrease in thermal conductivity, especially at 25% LF capacity, where it was about 0.105 W/m·°C [12].

Accordingly, the field is still open to study the impact of the mechanical properties of eco-friendly plaster on compression and tension with polyester resin polyester hardener. Specifically, this area has not been delved into in recent research. In this regard, this study seeks to conduct tensile and compression testing to determine the most important biomechanical properties, such as the maximum flexibility and quality, through studying eco-friendly plaster with polyester resin and polyester hardener. The novelty of this research is that it presents the effect of this mixture on the mechanical properties and its advantages.

## 2. Materials and Methods

This section presents the materials and methodology utilized in this research. It introduces the mixture materials used for the procedures of the mechanical tests. It includes the sample preparation, the procedures of the mechanical and emission tests, and a brief explanation for ANSYS software.

### 2.1. The Mixture Materials

A commercially available gypsum-based plaster with the following chemical makeup was the plaster material used in this study:

- H_2_O (water): 20–25% by weight;
- CaSO_4_ (calcium sulfate): 75–80% by weight;
- Additives: 0–5% by weight (such as retarders, accelerators, and fillers).

The plaster contained hemihydrate calcium sulfate (CaSO_4_·0.5H_2_O). The dimensions of the plaster particles varied, ranging from around 10 to 200 μm. The measured median particle size (D50) was 50 μm. The range of particle sizes within which it fell was between 30 and 80 μm. The majority of the plaster particles were angular in shape, with a rough surface, some porosity, and internal spaces. The plaster’s specific surface area was determined to be 2.8 ± 0.2 m^2^/g.

Plaster, polyester hardener, and polyester resin were the mixed materials employed in this investigation. Yamama Gypsum and Saudi Industrial Resins (SIR) are manufacturers of the material used in this study. Polyester resin is a thermoset polymer that is produced by polymerizing dihydric alcohols and dicarboxylic acids. Because of its cost-effectiveness, chemical resistance, and excellent mechanical qualities, it is widely employed in many different industries. The online retailer polyestershoppen.com, which specializes in offering premium polyester materials for industrial and do-it-yourself applications, is where the polyester resin and hardener utilized in this study were purchased.

### 2.2. Specimen Dimensions

For the compression test, cube-shaped specimens measuring 50 mm × 50 mm × 50 mm were selected. For the tension test, cylindrical specimens were chosen, each having a length of 100 mm and a diameter of 50 mm.

### 2.3. Sample Preparation

With an accuracy of ±0.01 g and a measuring range of up to 8200 g, the Sartorius Entris II balance was used. Three different resin ratios—30%, 45%, and 60%—were meticulously measured and combined with plaster, polyester hardener, and polyester resin for sample preparation. A high-precision scale was used to weigh the proper quantities of each component, which were then mixed in a sanitized container. To guarantee that the ingredients were distributed evenly, the mixture was vigorously mixed by hand for a number of minutes. To make the test specimens, the blended material was mixed and then poured into typical mold cavities. The non-stick substance used to make the molds durable made it easier to remove the cured samples. After that, the filled molds were put in a controlled environment for 72 h, where the polyester mixture was allowed to fully cure and harden at a temperature of 25 °C and 50% relative humidity.

### 2.4. Mixing Method

The following actions were carried out to guarantee complete and precise mixing of the ingredients during the polymerization process:

- The plaster was polymerized at a regulated temperature of 22 ± 2 °C after the constituent parts had been mixed.
- Five minutes was allotted for combining the plaster ingredients, giving enough time to guarantee a uniform mixture prior to the polymerization reaction commencing.

Table 1 presents the grams of polyester resin, polyester hardener, and plaster produced during the mixing process to produce each of three percentages (30, 45, and 60%). Thirty percent was the initial percentage that was put to work using 1295 g, 23 g, and 555 g for polyester resin, polyester hardener, and plaster, respectively. These grams were approximately (~69%), (~1.5%), and (~29.5%) of the total, respectively. Following process completion, the process at 45% was initiated with the components given in Table 1. Subsequently, a 60% concentration was prepared as listed in Table 1. To fill the resulting liquid in geometric forms to form it into the shape of a diamond or cylinder, the geometric shapes that the liquid will form must first be thoroughly cleansed. Twelve geometric shapes were assigned, including six from the cube and six from the cycle, for every percentage [13,14,15].

**Figure 1:**
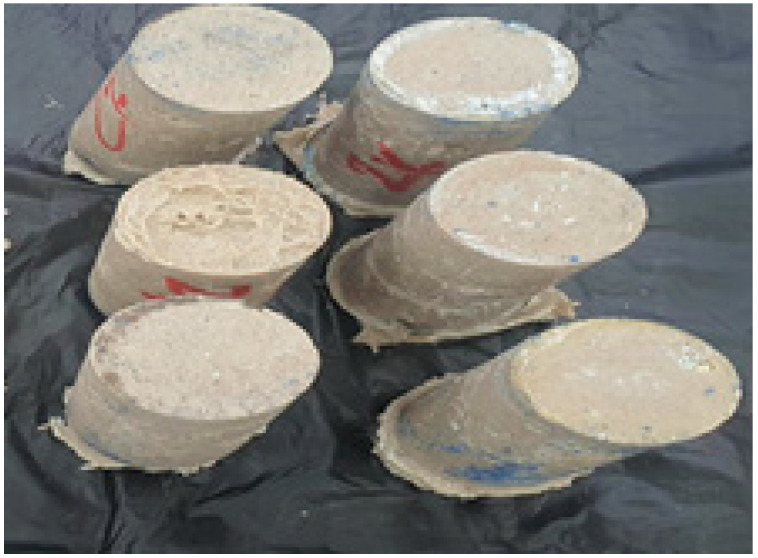
Samples prepared for mechanical test.

The components were mixed with a Bosch-type sort mixer. The size of the mixing batches and the amount of power needed determined which exact model was selected. To guarantee a completely uniform and well-blended material, the viscosity of the combination, as well as its color and texture, were checked during the mixing process. Before starting the casting process, the consistency must be smooth, constant, and pourable. The samples that were subjected to mechanical tests are presented in Figure 1. In this study, a number of samples were prepared and subjected to compression and tensile tests. These samples are ($N_{n}$), where n is the number of samples in each test, separately.

### 2.5. Compression Test

A universal testing machine (UTM) was used to determine the compressive strength of the polyester mixture. The device in question was the MTS Landmark 810 universal testing machine, which is produced in Eden Prairie, Minnesota, USA, by MTS Systems Corporation. A total of fifteen cube-shaped specimens underwent testing. Three distinct ratios of polyester hardener to resin were used to prepare these samples, including 30%, 45%, and 60%. Five distinct samples from each ratio group were put through the compression test. The compression process was completed at a constant speed of 1 mm/min.

Compression testing was conducted in compliance with ASTM D695 standard [16] test procedures for the compressive properties of rigid polymers at a deformation rate of 2 mm/min. The maximum compressive force capability of the MTS Landmark 810 UTM is 250 kN. For the compressive and tensile testing, the test specimens were placed into the UTM grips and deformed at a rate of 1 mm/min. In order to guarantee a quasi-static loading condition and enable adequate data collection throughout the experiments, this deformation rate was chosen.

In order to precisely measure and record the data during the experiments, the UTM was outfitted with the following sensors:

- Displacement transducers to track the test specimens’ deformation as they are being loaded.
- Load cells with a 100 kN maximum force capacity to measure the applied compressive and tensile forces.
- Humidity and temperature sensors to keep the surroundings within the designated range.

The polyester specimens were subjected to compressive force during the testing, and the resulting deformation (displacement) was measured and recorded [15].

### 2.6. Tensile Test

The tensile test used, as shown in Figure 1, was conducted on cylindrical-shaped specimens and used the splitting tensile test according to ASTM C496 [17] on the specimens. This standard is a definitive guide that everyone turns to in the processes of testing materials and the things that it processes and focuses on, which is the evaluation of the tensile strength by splitting for each of the specimens, especially cylindrical concrete. This method involves subjecting a cylindrical shape to a diagonal compressive force along the entire length of the sample until the point of failure is reached. The important and main aspect of conducting such a test is to stimulate tensile pressures at the level that contains the applied load, accompanied by high pressures. Relatively, in the immediate surrounding area, pressure is exerted on opposite sides of the sample to understand and know the material’s response to stress. This test is based on the amount of force resulting from the pressure applied to the sample. The concrete collapses due to tension instead of the resulting pressure, and tension can occur in the affected areas. Under the load, which suffers from triaxle compression, this allows it to withstand much higher pressures compared to a uniaxial compressive strength test. The results that appear through the test based on the ASTM C496 standard play an important and powerful role in the engineering and design of lightweight concrete structural parts; moreover, by knowing the tensile strength, the sample can be accessed according to the material, and engineers in general can make decisions and form ideas about the material’s performance under tension. ASTM C496 is much more than a test protocol. It has become the cornerstone for understanding the way concrete behaves under certain impacts and for working to develop it. It is possible to assess the variations in mechanical behavior by conducting tests in both compression and tension. When paired with the findings of the tensile test, the compressive strength statistics offer thorough knowledge of the material’s entire performance. By evaluating samples with hardener-to-resin ratios of 30%, 45%, and 60%, the best formulation for achieving the required balance of compressive and tensile qualities can be found [14,16,17,18].

### 2.7. Emission Test

The device used to determine the chemical makeup of the flue gases generated by industrial combustion operations was an industrial flue gas analyzer. Particulate matter (PM), sulfur dioxide (SO_2_), nitrogen oxides (NOx), carbon monoxide (CO_2_), and sulfur dioxide (SO_2_) can all be measured with this equipment. Continuous data collection was made possible by momentarily moving the thermocouple 2–3 cm closer to the combination in order to achieve exact adjustments when measuring a small mixture [14,18]. In order to ascertain whether any pollutants were released during the production process or while employing environmentally friendly plaster, emission tests were conducted. The USA-made Kane 988 Automotive Diagnostic Exhaust Gas Analyzer was utilized in this investigation. The emission testing assessed the gaseous byproducts released during the industrial processes but did not directly evaluate the product’s flammability or combustion characteristics. Measuring the gases released during material burning, such as carbon dioxide (CO_2_), sulfur dioxide (SO_2_), and nitrogen oxides (NOx), was the goal. Evaluating the plaster’s potential environmental impact and environmental friendliness when in use was the main goal. The gaseous emissions from materials, which include pollutants such as nitrogen oxides, sulfur dioxide, carbon monoxide, and carbon dioxide, were measured using the ASTM D6348 standard. Emissions from plaster were measured when it was heated to high temperatures [19,20,21,22,23].

### 2.8. ANSYS Software

The precision of our technical advancements was greatly increased by the capacity to represent the intricate and realistic aspects of materials using ANSYS software (Ansys 2024 R2). The purpose of this study was to replicate the tensile test using the same materials and dimensions as those used in the experimental investigation. The properties of density, modulus of elasticity, shear modulus, coefficient of thermal expansion, and geometric dimensions of the models (height, width, and length), as well as the model’s shape and the kinds and distribution of loads on it, were the input parameters used in the simulation. This simulation’s primary goal was to create a thorough comparison between the simulation’s output and the experimental curves that came from mechanical testing. The results of this comparison study supported and validated the conclusions drawn from our mechanical testing.

## 3. Results and Discussion

In this section, we aim to thoroughly explore and detail the complex mechanical properties of the material being studied, offering an in-depth understanding of its response to various types of loads. This segment also focuses on highlighting the key insights from an extensive series of compression and tensile tests. These tests, carefully planned and executed, are crucial for assessing how the material behaves when subjected to external forces.

Furthermore, this section includes a detailed analysis of the results from the emission tests. These tests were conducted to evaluate the material’s likelihood of emitting harmful substances. The comprehensive tests conducted—including compression, tensile, and emission tests—were applied to three different mixing ratios. Each ratio consisted of four test samples.

### 3.1. Compression Stress Results

**Figure 2:**
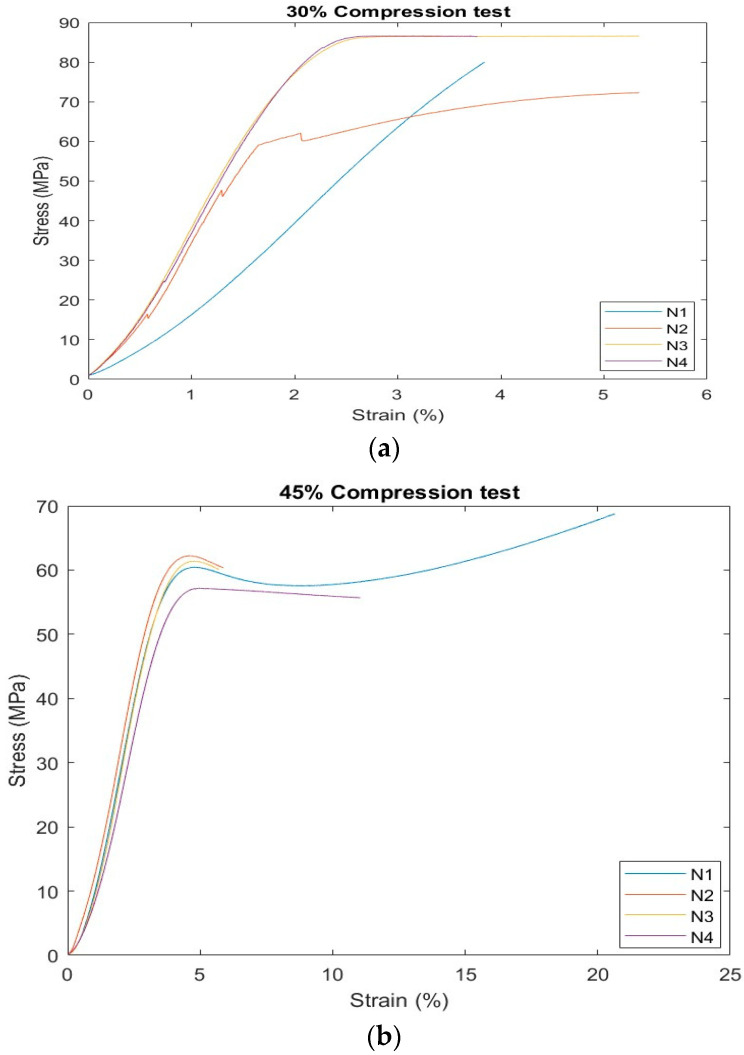

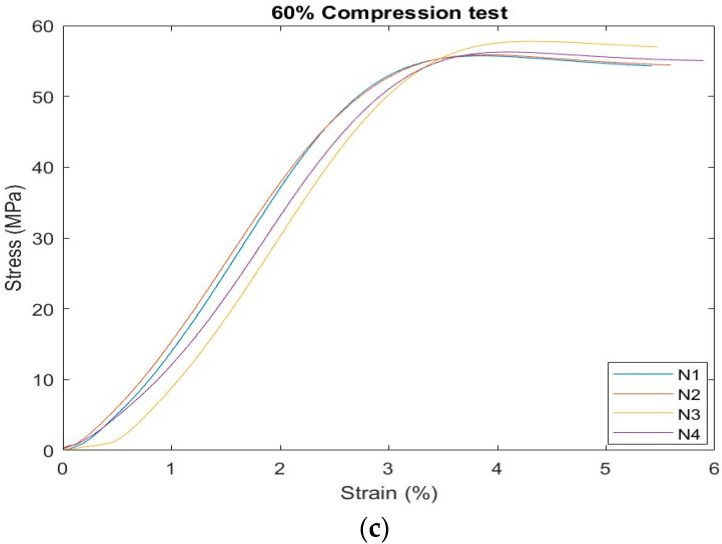
Stress–strain curve in compression test of BSCO Plaster (**a**) 30%, (**b**) 45%, and (**c**) 60%.

The findings suggests that, up until when the strain exceeds 2%, the material in question exhibits a constant and linear behavior in the stress–strain relationship. Following that, it appears as though the material reaches a saturation point, also known as the plastic limit, at which time the stress stabilizes and does not rise as the strain does. This difference in the mechanical behavior of the material samples may have an impact on their applications and final properties (see Figure 2a). In the compression test of 45%, the stress–strain curve shows that the stress increases as the strain percentage increases for four samples until it reaches the max stress at strain percentage 5%. Only the stress of sample N1, which represents one sample of five samples utilized for 1st ratio, as presented in the Supplementary Material section, is still increasing compared to that of the other samples, as shown in Figure 2b.

Figure 2c presents the relation between strain and stress for four used samples at 60% (compression test). The results show that the stress increased by strain with the same sequence for four samples until it reached strain 3.5%, where the stress became constant. This finding suggests that the material in question exhibits a constant and linear behavior in the stress–strain relationship. Following that, it appears as though the material reaches a saturation point, also known as the plastic limit, at which time the stress stabilizes and does not rise as the strain does.

To investigate the microstructure and quality of gypsum–polymer mixing, all of the sectional samples were analyzed. In the samples with 45 and 60% gypsum, it was found that the gypsum particles were dispersed uniformly throughout the polymer matrix. The microstructure showed no signs of significant abnormalities or agglomerations. The mixing quality was deemed excellent, and the gypsum particles were evenly dispersed. The microstructure of the samples with 30% gypsum revealed localized agglomerations of gypsum particles. Within the polymer matrix, the gypsum particles were not evenly dispersed and instead were clustered. The irregular distribution and gypsum particle agglomerations found in the polymer matrix could have caused these samples’ varying mechanical performances.

More than one sample was tested to ensure the accuracy and reliability of the results, and the overall behavior of the material was represented by taking the average of these results, which helps in making better decisions about the use of the material. The characteristics and outcomes that emerged during the compression test at mixing ratio percentage 30%, according to Table 1, are discussed. Figure 2a presents the relation between strain and stress for four used samples at 30% (compression test), including N1, N2, N3, and N4, as presented in Table S1. It shows that the stress increased by strain for two samples until it reached a strain of 2%, then the stress became constant. The strain (%) of the other two samples was increased by stress. In terms of the mechanical parameters that have been tested, such as yield strength, elongation at break, and resilience modulus (according to Table S1), samples 3 and 4 seem to behave similarly with compared to samples 1 and 2. Specimens 1 and 2 have a yield strength that is somewhat greater than that of specimens 3 and 4. Compared to specimens 3 and 4, specimens 1 and 2 have a substantially higher modulus of resilience. Specimens 1 and 2 have substantially more ductility than specimens 3 and 4. These variations in mechanical characteristics resulted from the variations in the production circumstances of the various samples.

Table 2 demonstrates how the mechanical characteristics of the samples are greatly impacted by the percentage of polyester. Thirty percent has a lower modulus of elasticity than the proportions with higher values, meaning that it has a lower load-bearing capability but a higher elasticity. The modulus of elasticity of the samples containing 45% and 60% polyester, on the other hand, was much greater; therefore, this indicates that the material is stiffer and less elastic at these amounts. However, because the changes were so tiny, there was no discernible gain in the ultimate tensile strength or yield strength between the various amounts. In order to give a thorough comparison between the simulation results and the experimental results for three percentages (30, 45, and 60%), both the average and the simulated experimental findings are presented. It became clear that there are slight differences between them, and they are considered within the acceptable limits. At the 30% ratio, the difference percentages between the simulated and experimental results were 50, 1, 3, 80, 7, and 55% for the modulus of elasticity, UTS, yield strength, yield strain, modulus of resilience, and ductility, respectively; 26, 0.1, 0.1, 1, 14, and 5%, respectively, at the 45% ratio; and 2, 1, 3, 0.6, 12, and 9% at the 60% ratio, respectivley. In terms of the mechanical performance, 30% is the best option, since it offers an excellent balance between stiffness and durability.

### 3.2. Tensile Stress Results

**Figure 3:**
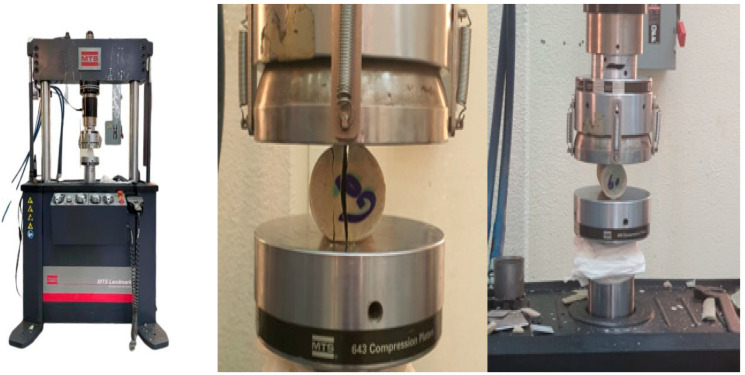
Tensile and compression testing device MTS and the sample (during) tensile test of Plaster 60%.

Following the procedures of the tensile strength test, as presented in Section 2.6, the results show the characteristics and behavior of the material used during the tensile test. Figure 3 shows the compression/tensile test machine and the sample under test. Figure 3 illustrates the MTS device and the sample (during) tensile test. Table 3 shows the mechanical properties (tensile test) of the gypsum–polyester mixture samples for the three different ratios (30%, 45%, and 60%). The table shows the values through the simulation and experimental average for samples of each ratio, according to Table S2. It is noted that, at 30%, according to the simulation results and experimental results, respectively, the modulus of elasticity was 1570.27 and 539.215 MPa, while the yield strength was 39.12 and 39.21 MPa, and the ductility was 3.85 and 3.555. At 45%, the modulus of elasticity was 1317.75 and 917.7 MPa, the yield strength was 29.81 and 29.975 MPa, and the ductility was 3.98 and 4.1825. These results differed significantly at 60%, where the elastic modulus was 1408.5 and 1255.2 MPa, while the yield strength was 28.3 and 28.4 MPa. In light of these results, it is clear that there is a discrepancy between the simulation results and the experimental results. In some cases, such as UTS and yield strength, the simulated and experimental values were close, indicating good accuracy in the simulation for these properties. However, there is a noticeable difference in the elastic modulus, where the simulation was much higher than the average values at all ratios, which means that the simulation may overestimate the stiffness of the material. The elastic modulus was much higher in the simulation compared to the experimental results, indicating that the material was more elastic in the experiments than in the simulation. In contrast, the ultimate tensile strength and yield strength were more consistent between the simulation and the experiments. Plasticity shows little variation, indicating reasonable agreement in the behavior of the material under deformation. The variation between the simulation and the experimental test outcomes could be the result of environmental variables such as temperature and humidity, which have distinct effects on the outcomes in the simulation and the actual test.

**Figure 4:**
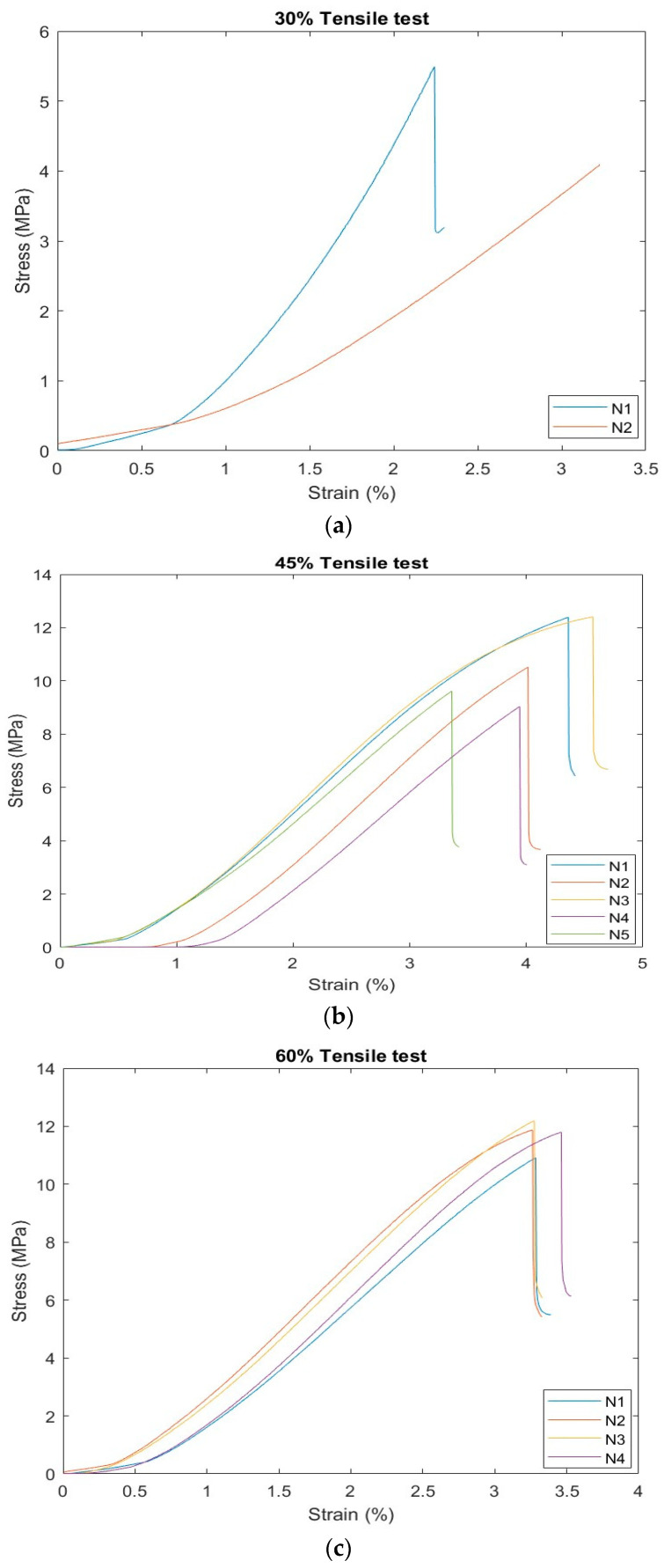
The stress–strain curves of tensile test of Plaster (**a**) 30%, (**b**) 45%, and (**c**) 60%.

Figure 4a shows the stress–strain curves of the tensile test of Plaster 30%. This result indicates changes in the internal structure of the plaster, such as cracks or deformations, resulting in the loss of the ability to withstand further stress for sample N2. With only two specimens, the sample size is too small to reliably determine the true or average values of the modulus and strength for the 30% filler material. It is evident that N1 is the superior choice. The load of N1 was 209.4 kN, the stress was 20.3 MPa, and the strain was 3.16%. Figure 4b shows the stress–strain curves of the tensile test of Plaster 45%. This illustrates that stress increases by strain for the test samples. Figure 4c shows the stress–strain curves from the Plaster 60% tensile test.

This result concluded that the mechanical behavior was the best at 30% ratio concentration when compared to the other ratios. Its fourth sample (N4) was the best among all of the samples, as shown by its ability to withstand a force of 215.9533 kN. Its elongation capacity reached 8.92684%, so it was considered the best; however, in the tensile test, it seems that N3 was the best choice, with a rate of 45%, including its ability to withstand high forces of 97.36592 kN, and the elongation coefficient was 4.65399%, which is considered a reasonable percentage. It is clear that the best ratio is 30%, as it shows higher values in all characteristics compared to the other ratios. This means that this material has the best mechanical properties at 30% loading.

The value known as the standard deviation measures how spread out the data are around the mean. In light of the sample results, there is a higher level of variation and more consistency, indicating improvement or stability in the data, as shown in Table 4.

**Figure 5:**
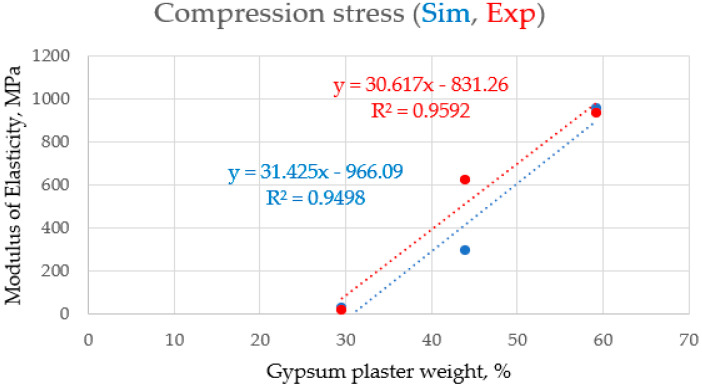
The consistency between the simulated and experimental data for modulus of elasticity (MPa) after compression tests.

**Figure 6:**
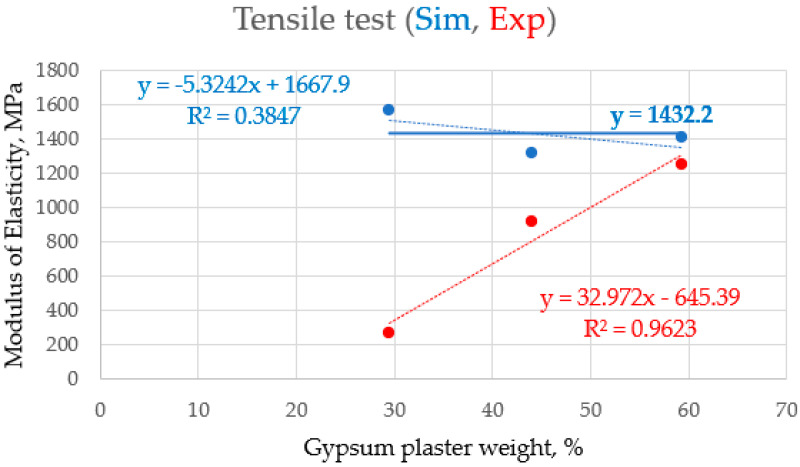
The consistency between the simulated and experimental data for modulus of elasticity (MPa) after tensile tests.

**Figure 7:**
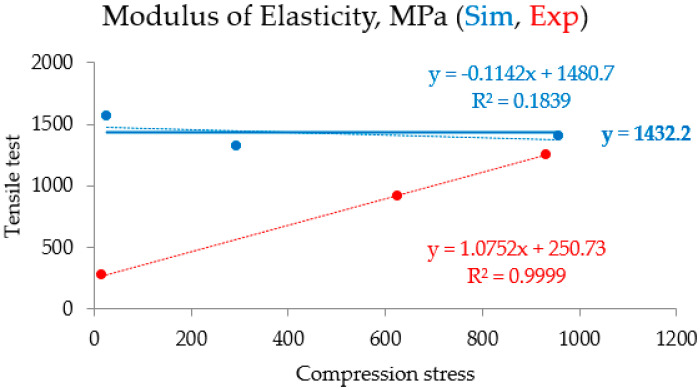
The consistency between the simulated and experimental data for compression and tensile tests.

The characteristics of the parameters measured (for the simulation and the actual experiment) after compression testing, rather than tensile testing, may be responsible for the greater consistency between the simulated and experimental data. This is because materials behave more consistently when compressed, but surface defects, or small discrepancies, can cause premature failure in tensile tests. Furthermore, since compression testing is not typically used, this can lead to more accurate readings. Furthermore, by carefully calibrating the simulation used in the compression tests, more accurate predictions were made that agreed better with the experimental results. The reason for the consistency between the simulated and experimental data for compression tests compared to tensile tests is that as the percentage of gypsum weight in the composite material increased, and the samples’ modulus of elasticity increased approximately linearly for both simulated and experimental points. These values agree well with each other at values of “~30%” and “~60%”, as well as at a value of “~45%”. The data types showed a significant disparity, as illustrated in Figure 5. Figure 6 and Figure 7 indicate that, for all testing points (“~30%”, “~45%”, and “~60%”), the modulus of elasticity of the samples increased almost linearly with an increase in the percentage of gypsum weight in the composite material. But still, regardless of the examined point and the gypsum weight percentage within the composite material, the elasticity modulus of the samples does not vary with the weight percentage of gypsum in the composite material. This R2 data value of 0.3847, with the extremely low indication, implies that data correlation does not exist in this case. Therefore, fairly satisfactory results model stretching samples and derive their mechanical properties from modeling compression processes in the ANSYS program, which is designed for the mechanical simulation of composite materials.

### 3.3. Emissions Test

The goal of this research is to lower emissions into the environment. To ensure that we are headed in the correct direction, an emission test was carried out every five to sixty minutes. The test site was set up to guarantee height and parameter stability. Carbon dioxide, sulfur dioxide, and nitrogen dioxide were the gases put to the test. As indicated in Table 5, it was carried out in the following three ratios: 30%, 45%, and 60%. Parts per million (ppm) of carbon dioxide (CO_2_), sulfur dioxide (SO_2_), and nitrogen dioxide (NO_2_) were among the measured emissions. Different time intervals (0, 15, 30, 45, and 60 min) were used to take the measurements. The findings demonstrate that, for all three pollutants (CO_2_, SO_2_, and NO_2_), the emission levels were typically modest over a range of filler percentages and time intervals. For all three contaminants, the emissions were minimal or nonexistent when using the 30% filler. There were some non-zero emission values for the 45% and 60% filler, although they were still rather low, especially for SO_2_. These results conform to the standard for the three mixing percentages. It can be concluded that the substance or system under test has a relatively low environmental impact in terms of the assessed air pollutants based on the low emission levels recorded. The findings of the emission test show that the material under test performs well in terms of the environment, with low concentrations of the air pollutants being measured. Overall, in terms of the mechanical tests, the 30% ratio outperformed the 45% and 60% ratios, according to the data.

## 4. Conclusions

This research aims to study the benefits of gypsum in terms of mechanical properties when combined with polyester resin and hardener under pressure and tension—especially since it is an environmentally friendly material—in an attempt to develop this material for use in different applications in addition to taking into account its impact on the environment. In this regard, the use of different ratios of components (30%, 45%, and 60% of gypsum plaster weight) were studied in order to choose the optimal ratio, both economically and environmentally, in terms of mechanical properties, in addition to preserving the environment by determining the gases emitted during the emission test. The mechanical properties and emission test of three samples with different ratios of components (30%, 45%, and 60% of gypsum plaster weight) were studied in order to find the optimal ratio mechanically, economically, and environmentally. By combining gypsum, polyester resin, and polyester hardener, we were able to determine the emitted gases. It is also noted that there was no carbon dioxide in any quantity, which is a positive indicator. However, sulfur dioxide and nitrogen dioxide were detected, and the highest emission rates for sulfur dioxide and nitrogen dioxide reached 2, 3, 0, and 5.3.16, respectively, at 45% of gypsum plaster weight. The lowest emission rates were observed in the 60% and 30% samples. On the other hand, when conducting mechanical tests, such as compression and tension, via simulation on the ANSYS program and comparing them with laboratory results, it was found that gypsum, when combined with polyester resin and hardener at a rate of 30%, is the most suitable and beneficial option when considering four samples with the same ratio. This study recommends using a ratio of 30%, according to the main mechanical properties, comprehensively, which confirms that it is the best mechanically, as well as economically.

## Figures and Tables

**Table 1:** Mixing ratios of the materials, including polyester resin, polyester hardener, and plaster (%).

Title of Ratio (Percent of Plaster)	30%	45%	60%
Polyester resin	1295 g (~69%)	1018 g (~55%)	740 g (~40%)
Polyester hardener	23 g (~1.5%)	18 g (~1%)	13 g (~0.7%)
Plaster	555 g (~29.5%)	832 g (~44%)	1110 g (~59.3%)
Cube and cylinder	6 and 6	6 and 6	6 and 6

**Table 2:** Comparison between simulation and experimental (average) results for mechanical properties after applying compression stress.

Title		Modulus of Elasticity (MPa)	UTS (MPa)	Yield Strength (MPa)	Yield Strain (mm)	Modulus of Resilience (kJ/m^3^)	Ductility
30%	Simulation	24.74	63.18	56.82	0.031	0.868	10.85
	Experimental	16.16	62.82	58.20	0.195	0.820	5.000
45%	Simulation	792.4	57.62	55.80	0.029	0.809	16.69
	Experimental	624.6	57.71	55.67	0.028	0.772	17.10
60%	Simulation	957.9	56.82	55.79	0.023	0.796	12.38
	Experimental	931.5	57.26	53.90	0.029	0.829	11.84

**Table 3:** Comparison between simulation and experimental (average) results for mechanical properties after applying tensile test.

Title		Modulus of Elasticity (MPa)	UTS (MPa)	Yield Strength (MPa)	Yield Strain (mm)	Modulus of Resilience (kJ/m^3^)	Ductility
30%	Simulation	1570.3	41.87	39.12	0.029	0.579	3.850
	Experimental	269.61	21.09	19.61	0.018	0.382	1.778
45%	Simulation	1317.8	34.90	29.81	0.030	0.400	3.980
	Experimental	917.70	35.48	29.98	0.033	0.428	4.183
60%	Simulation	1408.5	33.8	28.30	0.030	0.370	4.260
	Experimental	1255.2	33.65	28.40	0.038	0.340	3.980

**Table 4:** Standard deviation for sample results after applying mechanical tests.

Title	Compression Test			Tensile Test		
	30%	45%	60%	30%	45%	60%
Modulus of Elasticity (MPa)	14.5	695.7	833.59	304.79	285.58	1121.88
UTS (MPa)	53.8	51.0	49.75	29.50	27.39	29.55
Yield Strength (MPa)	49.5	49.0	47.66	26.75	16.64	24.78
Yield Strain (mm)	0.3	0.02	0.03	0.019	42.93	0.032
Modulus of Resilience (kJ/m^3^)	0.7	0.68	0.77	0.51	77.56	0.324
Ductility	4.3	13.02	10.21	2.47	40.049	3.591

**Table 5:** Emission test comparison.

Time (min)	30%			45%			60%		
	CO_2_ (ppm)	SO_2_ (ppm)	NO_2_ (ppm)	CO_2_ (ppm)	SO_2_ (ppm)	NO_2_ (ppm)	CO_2_ (ppm)	SO_2_ (ppm)	NO_2_ (ppm)
0	0	0	0	0	0	0	0	0	0
15	0	0	31	0	2	5	0	1	0
30	0	0	12	0	3	3	0	1	16
45	0	0	0	0	0	16	0	2	7
60	0	0	0	0	0	0	0	0	0

## Data Availability

The datasets used during the current study are available from the corresponding author on reasonable request (due to privacy).

## Supplementary Materials

The following supporting information can be downloaded at: https://www.mdpi.com/article/10.3390/polym16192847/s1, Table S1: Mechanical properties in compression tests of three plaster ratios (%). Table S2: Mechanical properties in tensile tests of Plaster 30%, 45%, and 60%.
