# Supplementary material for: Benefits of Environmentally Friendly Plaster on Mechanical Properties When Combined with Polyester Resin and Hardener Are Examined under Compression and Tension
Source: Polymers (Basel). 2024 Oct 9;16(19):2847. doi: 10.3390/polym16192847 (PMC11478348; doi:10.3390/polym16192847)
Supplement: Supplementary file 1 [file polymers-16-02847-s001.zip › polymers-3177228-supplementary.pdf]

polymers

MDPI

Supplementary Material

This section introduces Supplementary Material used in this research. It included Modulus of Elasticity (MPa), UTS (MPa), Yield Strength (MPa), Yield Strain (mm), Modulus of Resilience (kJ/m3), and Ductility for all samples subjected to compression and tensile tests. Table S1 shows the mechanical properties for used samples N1, N2, N3, and N4 after applying compression test for materials of 30%, 45%, and 60% ratios, respectively.

Table S1. Mechanical properties in compression tests of three plaster ratios (%).

Title		Modulus of Elasticity (MPa)	UTS (MPa)	Yield Strength (MPa)	Yield Strain (mm)	Modulus of Resilience (kJ/m³)	Ductility
30%	Simulation	24.74	63.18	56.82	0.031	0.868	10.85
	N1	15.69	64.99	61.47	0.032	0.984	5.13
	N2	11.54	62.88	60.77	0.035	0.999	5.22
	N3	16.67	61.77	55.77	0.355	0.754	5.39
	N4	20.73	61.62	54.78	0.357	0.544	4.25
	Experimental (Average)	16.16	62.82	58.20	0.195	0.820	5.000
45%	Simulation	792.37	57.62	55.79	0.029	0.809	16.69
	N1	313.64	54.23	53.06	0.028	0.731	27.37
	N2	857.14	59.34	55.16	0.026	0.727	15.03
	N3	998.00	59.41	59.23	0.030	0.892	15.11
	N4	329.41	57.87	55.23	0.027	0.739	10.90
	Experimental (Average)	624.6	57.71	55.67	0.028	0.772	17.10
60%	Simulation	957.87	56.82	55.79	0.023	0.796	12.38
	N1	850.00	56.72	50.67	0.028	0.659	11.99
	N2	866.67	57.91	56.78	0.038	0.790	12.37
	N3	1045.55	56.97	52.82	0.023	0.890	11.59
	N4	963.64	57.45	55.34	0.025	0.976	11.39
	Experimental (Average)	931.5	57.26	53.90	0.029	0.829	11.84

Table *S*2 presents the mechanical properties of plaster under tensile stress for three different ratios. For the 30% ratio, it is observed that stress increases with strain for the test samples. However, for sample N1, stress begins to decrease after reaching 2.2% strain and 5.5 MPa stress. For the 45% ratio, stress consistently increases with strain across all test samples, with sample N1 demonstrating superior performance. Sample N3 exhibits a load of 97.4 kN, stress of 45.84 MPa, and strain of 4.65%. For the 60% ratio, sample N3 is identified as the better performer, with sample N4 showing a load of 95.63 kN, stress of 45.03 MPa, and strain of 3.59%.

Table S2. Mechanical properties in tensile tests of Plaster 30%, 45%, and 60%.

Title		Modulus of Elasticity (MPa)	UTS (MPa)	Yield Strength (MPa)	Yield Strain (mm)	Modulus of Resilience (kJ/m³)	Ductility
30%	Simulation	1570.27	41.87	39.12	0.029	0.579	3.85
	N1	666.67	42.63	40.61	0.060	0.814	3.62
	N2	411.76	41.71	37.81	0.013	0.715	3.49
	N3	0	0	0	0	0	0
	N4	0	0	0	0	0	0
	Experimental (Average)	269.61	21.09	19.61	0.018	0.382	1.778

**45%**	Simulation	1317.75	34.90	29.81	0.03	0.40	3.98
	N1	918.4	35.56	28.28	0.03	0.38	4.24
	N2	1000.0	36.79	30.32	0.03	0.45	3.61
	N3	478.7	33.68	30.65	0.04	0.39	4.71
	N4	1273.7	35.88	30.65	0.03	0.49	4.17
	Experimental (Average)	917.70	35.48	29.98	0.033	0.428	4.183
**60%**	Simulation	1408.5	33.8	28.3	0.03	0.37	4.26
	N1	1142.9	32.3	27.8	0.04	0.27	3.61
	N2	1363.6	33.8	27.6	0.03	0.35	3.48
	N3	1285.7	35.1	28.6	0.04	0.27	4.00
	N4	1228.6	33.4	29.6	0.04	0.47	4.83
	Experimental (Average)	1255.2	33.65	28.40	0.038	0.340	3.980
